# How Metabolic State May Regulate Fear: Presence of Metabolic Receptors in the Fear Circuitry

**DOI:** 10.3389/fnins.2018.00594

**Published:** 2018-08-27

**Authors:** Lisa L. Koorneef, Marit Bogaards, Marcel J. T. Reinders, Onno C. Meijer, Ahmed Mahfouz

**Affiliations:** ^1^Division of Endocrinology, Department of Internal Medicine, Leiden University Medical Center, Leiden University, Leiden, Netherlands; ^2^Einthoven Laboratory for Experimental Vascular Medicine, Leiden University Medical Center, Leiden University, Leiden, Netherlands; ^3^Leiden Computational Biology Center, Leiden University Medical Center, Leiden University, Leiden, Netherlands; ^4^Delft Bioinformatics Laboratory, Delft University of Technology, Delft, Netherlands; ^5^Leiden Institute for Brain and Cognition, Leiden University Medical Center, Leiden University, Leiden, Netherlands

**Keywords:** energy balance, obesity, feeding, metabolism, fear, anxiety, fear circuitry, monoamines

## Abstract

Metabolic status impacts on the emotional brain to induce behavior that maintains energy balance. While hunger suppresses the fear circuitry to promote explorative food-seeking behavior, satiety or obesity may increase fear to prevent unnecessary risk-taking. Here we aimed to unravel which metabolic factors, that transfer information about the acute and the chronic metabolic status, are of primary importance to regulate fear, and to identify their sites of action within fear-related brain regions. We performed a *de novo* analysis of central and peripheral metabolic factors that can penetrate the blood–brain barrier using genome-wide expression data across the mouse brain from the Allen Brain Atlas (ABA). The central fear circuitry, as defined by subnuclei of the amygdala, the afferent hippocampus, the medial prefrontal cortex and the efferent periaqueductal gray, was enriched with metabolic receptors. Some of their corresponding ligands were known to modulate fear (e.g., estrogen and thyroid hormones) while others had not been associated with fear before (e.g., glucagon, ACTH). Additionally, several of these enriched metabolic receptors were coexpressed with well-described fear-modulating genes (*Crh, Crhr1*, or *Crhr2*). Co-expression analysis of monoamine markers and metabolic receptors suggested that monoaminergic nuclei have differential sensitivity to metabolic alterations. Serotonergic neurons expressed a large number of metabolic receptors (e.g., estrogen receptors, fatty acid receptors), suggesting a wide responsivity to metabolic changes. The noradrenergic system seemed to be specifically sensitive to hypocretin/orexin modulation. Taken together, we identified a number of novel metabolic factors (glucagon, ACTH) that have the potential to modulate the fear response. We additionally propose novel cerebral targets for metabolic factors (e.g., thyroid hormones) that modulate fear, but of which the sites of action are (largely) unknown.

## Introduction

Metabolic status affects the emotional brain to maintain energy balance. To this end, hunger facilitates explorative and food-seeking behavior via suppression of the fear circuitry (Levy et al., 2013; Burnett et al., 2016; Verma et al., 2016). Satiety and obesity – as opposite metabolic states – may increase fear levels to prevent unnecessary risk-taking, which could potentially progress to anxiety. Indeed, prolonged high-fat diet feeding in rodents enhanced fear-related behavior (Krishna et al., 2016). In humans, obesity increases the odds for a co-morbid anxiety disorder with 1.40 (CI 1.23–1.55) (Gariepy et al., 2010). Conversely, weight loss induced by bariatric surgery reduces anxiety scores, which is directly proportional to the amount of weight loss (de Zwaan et al., 2011; Edwards-Hampton et al., 2014; Kalarchian et al., 2015).

Mammals possess a wide range of signaling molecules, or “metabolic factors,” that provide cues about energy stores and impact on neurobiological processes to evoke an appropriate response. For example, neuropeptide Y (NPY) and its receptors play a key role in linking information from the gut (e.g., via pancreatic polypeptide) to food intake, energy homeostasis, anxiety and mood (Holzer et al., 2012; Verma et al., 2016). Similarly, gut-derived glucagon-like peptide 1 (GLP-1) regulates glucose homeostasis, food intake and anxiety behavior (Moller et al., 2002; Skibicka and Dickson, 2013; Anderberg et al., 2015).

Metabolic factors, such as NPY and GLP-1, can directly affect the fear circuitry, as formed by the subnuclei of the amygdala (the primary fear circuitry), the medial prefrontal cortex and the hippocampus (two major brain afferents), and the efferent periaqueductal gray (PAG, as a major output structure) (Kinzig et al., 2003; Verma et al., 2016). In the context of a positive energy balance, the monoaminergic nuclei are most often reported to be involved in the elevation of fear. The dopaminergic reward circuitry is often implicated in obesity for the reinforcing effects of ‘palatable’ food and is known to influence fear (Wang et al., 2001; Volkow et al., 2012; Abraham et al., 2014; Krishna et al., 2015; Morris et al., 2015). Less is known about the involvement of the noradrenalin (NE) system, but altered NE metabolism in hippocampus and prefrontal cortex has been associated with fear behavior in diet-induced obese mice (Krishna et al., 2015). Central serotonin (5-HT) signaling is most often reported as affected by obesity (Kimbrough and Weekley, 1984; Muldoon et al., 2006; Herrera-Marquez et al., 2011; Kurhe and Mahesh, 2015; Kurhe et al., 2015). Interestingly, a long high-fat diet intervention time of 36 weeks in mice, reflecting the human situation, increased fear in association with altered metabolism of 5-HT, but not of NE or dopamine (DA) (Krishna et al., 2016).

In this study, we aim to identify putative metabolic factors that influence fear, and to define where known factors may impinge on the fear circuitry. We performed a *de novo* analysis by characterizing receptor expression of metabolic factors in transcriptome data across the mouse brain as available through the Allen Brain Atlas (ABA) (Lein et al., 2007). We focused on mRNA expression of ‘metabolic receptors,’ i.e., receptors of metabolic factors, in the monoaminergic nuclei, as well as in the amygdala itself, the hippocampus, the medial prefrontal cortex, and the periaqueductal gray.

## Materials and Methods

### Selection of Candidate Metabolic Factors

We generated an extensive list of candidate metabolic factors that are involved in transferring information about the acute and the chronic metabolic state (**Table 1**). The evaluated central metabolic factors were the hypothalamic peptides and opioids that are part of, or affect the feeding regulation center (e.g., α-melanocyte stimulating hormone, orexin). Peripheral metabolic factors belonged to one of the following categories: (1) adipokines (e.g., leptin, visfatin) (2) nutrients (e.g., fatty acids) (3) gastro-intestinal hormones (GLP-1, ghrelin) (4) pancreatic hormones (glucagon, insulin) (5) hormones being part of endocrine systems that regulate metabolism [Hypothalamus-Pituitary-Adrenal-axis, Hypothalamus-Pituitary-Thymus (HPT-) axis, Growth hormone/insulin-like growth factor-1 axis] and (6) sex hormones (estrogens, androgens). The last category may be arguable, but was included as metabolic state affects estrogen and androgen levels to regulate physiology, reproductive status and related behavioral reactivity.

**Table 1:** Candidate metabolic factors and their receptors.

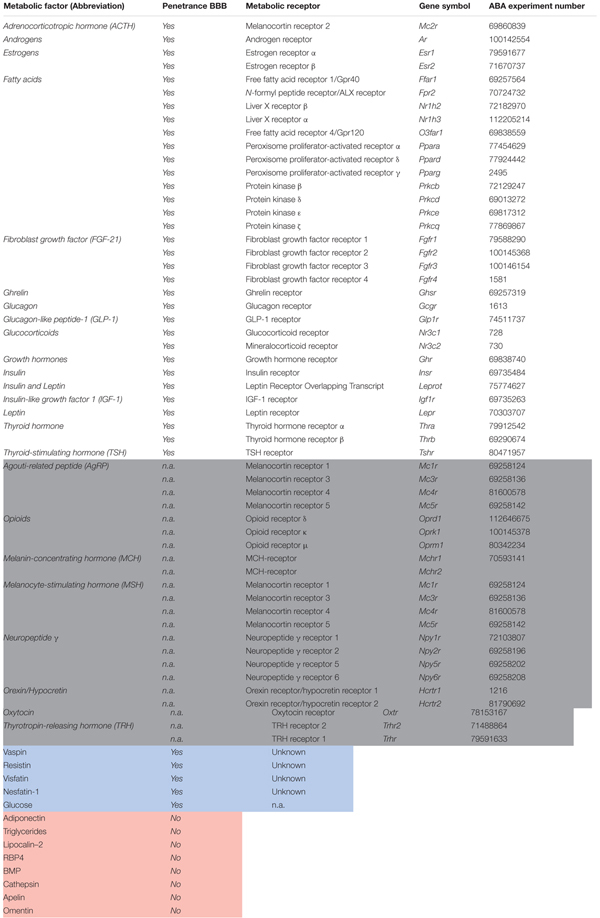

### Literature Research

We reviewed the literature on a direct causal relationship between ligands of metabolic receptors that were overrepresented in fear-related brain regions (**Supplementary Tables S1**–**S4**), and fear responses, and their putative sites of interaction, using PubMed as search engine (**Supplementary Table S5**). If the first 200 results did not show any relevant papers, we considered a causal relationship as ‘not found in literature.’ We solely included studies demonstrating a causal relationship between metabolic factor and fear, as found by micro- or intracerebroventricular injections or intranasal administration of receptor (ant-)agonists, full or conditional knock-out rodent models, overexpression models, or human polymorphisms, but not peripherally administered compounds.

### Selection of Fear-Related Brain Regions

**FIGURE 1:**
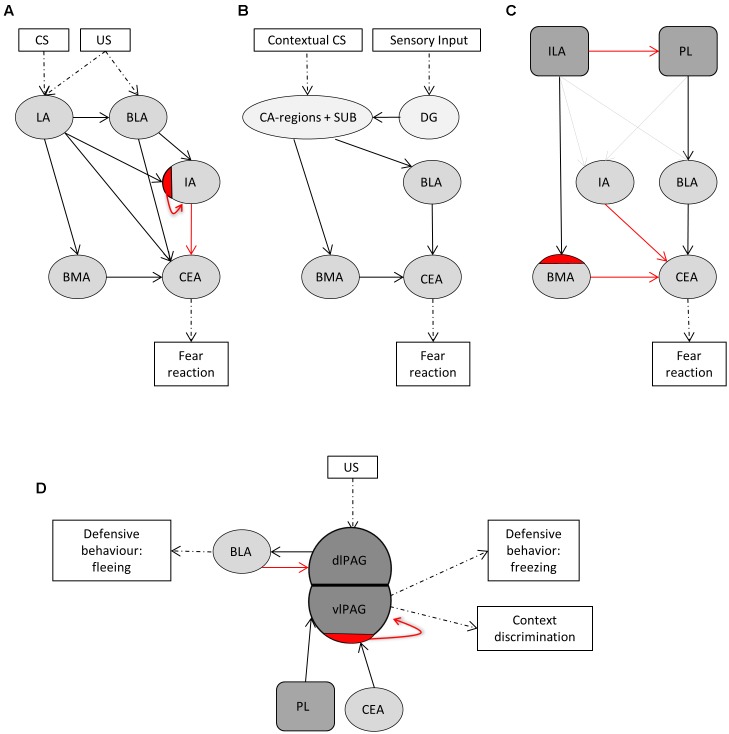
The central fear circuitry consists of subnuclei of the amygdala, the hippocampus, the medial prefrontal cortex and the efferent periaqueductal gray. **(A)** The lateral amygdala (LA) forms the main input and the central amygdala (CEA) the main output region of the amygdala. Integration of unconditioned (US) and conditioned (CS) stimuli takes place in the LA. From there on, fear signals are transferred via the basolateral amygdalar nucleus (BLA), basomedial amygdalar nucleus (BMA) and intercalated region (IA) to the CEA. Projections from the LA go to an inhibitory subdivision of the IA to disinhibit the CEA. **(B)** Information about the context of a fearful situation is transferred via hippocampal CA-regions and subiculum (SUB) to the BLA and to BMA and from thereon further to the CEA. The dentate gyrus (DG) was additionally included, as many sensory inputs travel via the DG before reaching the CA-regions. **(C)** The ventromedial prefrontal cortex (ILA) and the dorsomedial prefrontal cortex (PL) are important in the top-down control of fear and exert opposing effects. Activation of the PL leads to anxiogenesis via projections to the BLA, and, to a lesser extent, to the IA. Conversely, ILA activation leads to anxiolysis mainly via projections to inhibitory interneurons in the BMA, and via smaller projections to the IA and BLA. **(D)** The PAG is classically viewed as major output structure of the fear reaction, driving fear-related defensive behavior. Stimulation of the dorsolateral PAG serves as effective unconditioned stimulus for fear conditioning, and projections to the BLA evoke motor activity bursts that underlies fleeing behavior. The ventrolateral PAG receives inhibitory projections from the CEA, which disinhibits excitatory PAG neurons that project to motor nuclei to establish freezing behavior. The ventrolateral PAG additionally receives input from the PL, and these neurons control contextual fear discrimination. *Dashed arrows* indicate where stimuli enter or leave the fear circuitry. *Solid black arrows* reflect big excitatory while *solid gray arrows* reflect small excitatory projections. *Solid red arrows* indicate inhibitory projections and *red half-moons* inhibitory cell populations within a given subnucleus.

The central fear circuitry consists, among others, of subnuclei of the amygdala, the hippocampus, the medial prefrontal cortex, and the PAG. According to LeDoux’s model, the lateral amygdala (LA) forms the main input and the central amygdala (CEA) the main output region of the amygdala (**Figure 1A**) (LeDoux, 2000; Rodrigues et al., 2009). Integration of unconditioned and conditioned stimuli takes place in the LA. From there on, fear signals are transferred via the basolateral amygdalar nucleus (BLA), basomedial amygdalar nucleus (BMA), and intercalated region (IA) to the CEA. Projections from the LA go to an inhibitory subdivision of the IA to disinhibit the CEA (Pare et al., 2004). Information about the context of a fearful situation is transferred via hippocampal CA-regions and subiculum to the BLA and to BMA and from thereon further to the CEA (**Figure 1B**) (LeDoux, 2000). We additionally included the dentate gyrus, as many sensory inputs travel via the dentate gyrus before reaching the CA-regions. The ventromedial prefrontal cortex (ILA) and the dorsomedial prefrontal cortex (PL) are important in the top-down control of fear and exert opposing effects (**Figure 1C**) (Vidal-Gonzalez et al., 2006; Adhikari et al., 2015). Activation of the PL is anxiogenic via projections to the BLA (Vertes, 2004; Vidal-Gonzalez et al., 2006; Giustino and Maren, 2015), and, to a lesser extent, to the IA (Adhikari et al., 2015). Conversely, ILA activation is anxiolytic mainly via projections to inhibitory interneurons in the BMA and via smaller projections to the IA and BLA (Vidal-Gonzalez et al., 2006; Adhikari et al., 2015; Bloodgood et al., 2018). The PAG is classically viewed as major output structure of the fear reaction, driving fear-related defensive behavior. This is indeed the case for the ventrolateral PAG, but recent evidence shows that the dorsolateral PAG lies upstream of the amygdala. Stimulation of the dorsolateral PAG serves as effective unconditioned stimulus for fear conditioning, and projections to the BLA evoke motor activity bursts that underlies fleeing behavior (Brandao et al., 2008; Kim et al., 2013). The ventrolateral PAG receives inhibitory projections from the CEA, which disinhibits excitatory PAG neurons that project to motor nuclei to establish freezing behavior (**Figure 1D**) (Rizvi et al., 1991; McDannald, 2010; Tovote et al., 2016). The ventrolateral PAG additionally receives input from the PL, and these neurons control contextual fear discrimination (Rozeske et al., 2018).

### Determination of Gene Expression Levels in the Allen Brain Atlas

The Allen Mouse Brain Atlas (ABA) is a database of genome-wide expression data obtained by *in situ* hybridization experiments (Lein et al., 2007). Expression of the receptors of metabolic factors (**Table 1**) was determined based on absolute and normalized expression in the ABA. In short, we used the 3D-spatial expression volume of every gene in which expression measurements from *in situ* hybridization (ISH) experiments are summarized in isotropic voxels (200 μm), labeled with their anatomical nomenclature according to the ABA reference atlas. The absolute expression of each gene is represented by the expression energy, which is a combined measure of both expression level (the integrated amount of signal within each voxel) and the expression density (the amount of expressing cells within each voxel). We refer to the raw expression energy as the *absolute expression*. To account for the variable levels of expression between different genes, we calculated the *normalized expression* as the average expression of each gene in a brain region of interest divided by the average expression of that gene in the whole brain. We used Pearson’s correlation to assess the spatial co-expression relationships between pairs of genes based on the similarity of their 3D-expression pattern within a given region of the brain. To assess the strength of co-expression between a given set of genes (e.g., metabolic receptors) and a seed gene, we used a one-sided Wilcoxon rank-sum test [29]. The one-sided test means that we only focus on strong positive correlations.

## Results

### Selection of Candidate Metabolic Regulatory Factors

**FIGURE 2:**
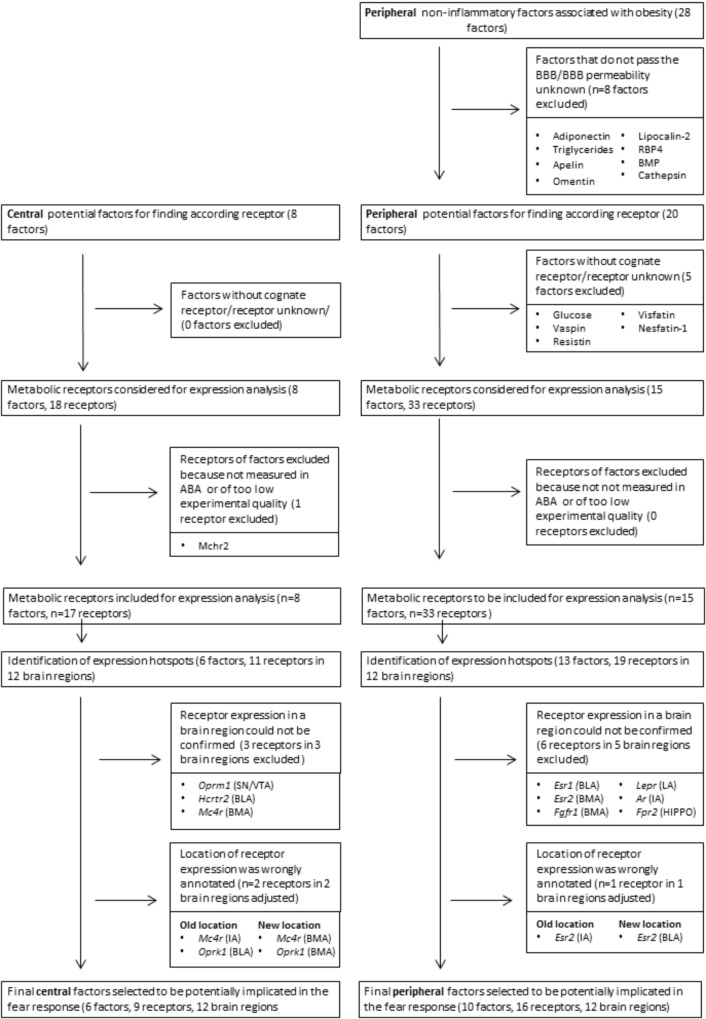
Flowchart of selection process of metabolic factors and their receptors. BBB, blood–brain-barrier; ABA, Allen Brain Atlas. Names of all other brain regions and metabolic receptors can be found in **Tables 1**, **2**.

A metabolic factor should possess two main characteristics in order to influence fear behavior: first, it should *reach* the brain and second, the fear-related brain region should be *responsive* to the factor. Following this reasoning, we made a preselection of metabolic factors that had the potential to affect fear. We initially included 28 peripheral and 8 central factors that transfer information about the acute or the structural metabolic status (**Table 1**). From this list, we excluded peripheral factors that do not cross the blood–brain barrier (*n* = 8, **Figure 2** and **Table 1**). The ability of a brain region to respond to a specific factor depends on whether the brain region expresses corresponding receptor(s). This led to the exclusion of all metabolic factors that did not have a known or cognate receptor (*n* = 5, **Figure 2** and **Table 1**). We next evaluated whether it was possible to measure expression of corresponding metabolic receptors. Metabolic receptors that had not been measured in the ABA or of which the experimental data were of insufficient quality were excluded (*n* = 1, **Figure 2** and **Table 1**). In this way a list of metabolic receptors (50 receptors of 23 factors, **Figure 2** and **Table 1**) was generated that was used for further receptor gene expression analysis.

### Metabolic Receptor Expression in the Amygdala and Higher Afferents

**FIGURE 3:**
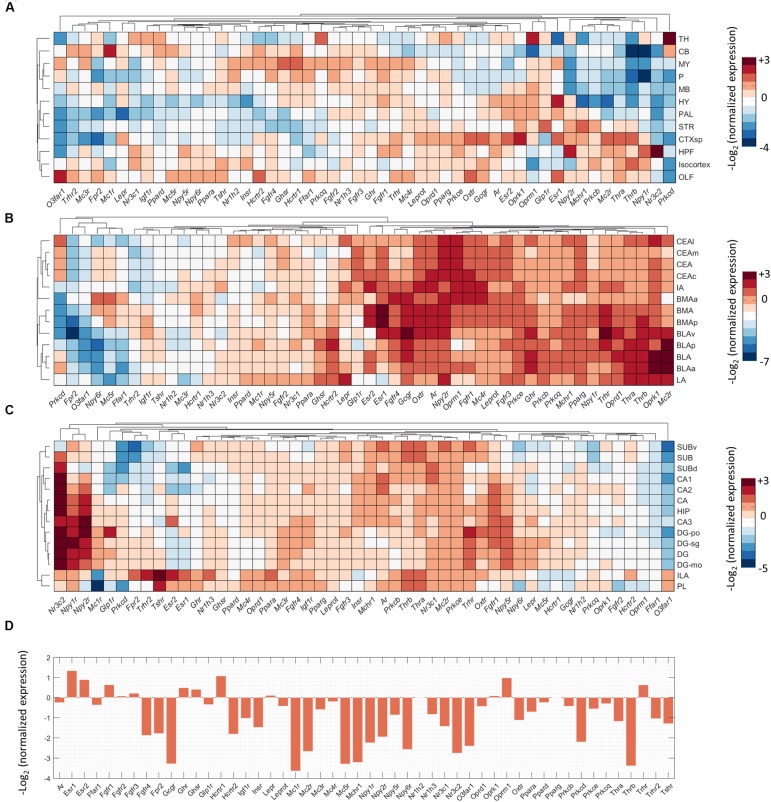
Expression summary of metabolic receptor genes in fear-related brain regions. Clustered normalized expression energies across: **(A)** 12 crude brain regions, **(B)** amygdala, **(C)** hippocampus and medial prefrontal cortex, and **(D)** periaqueductal gray. We calculated the normalized expression as the average expression of each gene in a brain region of interest divided by the average expression of that gene in the whole brain.

To investigate direct interactions of metabolic factors with the central fear circuitry (**Figure 1**), we mapped the expression of metabolic receptor genes in the subnuclei of the amygdala, hippocampus, medial prefrontal cortex and the PAG. Almost all metabolic receptors that were considered for expression analysis were expressed in at least one of the fear-associated brain regions (**Supplementary Figure S1**). Some receptors were in general highly expressed across all regions (e.g., *Prkb/c/e, Pparg*), whereas others showed a more selective expression pattern (e.g., *Thrb, Npy1r*). To avoid dominance of highly expressed genes and to allow detection of regional differences in expression energy of weakly expressed genes, we mapped the normalized expression of each gene with respect to the average expression of the gene in the whole mouse brain (**Figure 3**).

We observed that low normalized expression levels were not fully informative, as moderately expressed genes can have biological significance. For example, in the amygdala the glucocorticoid receptor (*Nr3c1)* is known to strongly affect the fear response but it is relatively underrepresented in the amygdala as compared to, e.g., hippocampus, cortex and thalamus (Kolber et al., 2008; Mahfouz et al., 2016). To select potential fear-modulating candidates, we therefore defined ‘expression hotspots’: regions where normalized expression levels of metabolic receptors were particularly high [i.e., log_2_(normalized expression) > 2 for amygdala, hippocampus and PFC, >1 for PAG, which is less neuron dense]. While regions with relatively low normalized expression may represent false negatives, these hotspots likely reflect true positives: areas that can be regulated by metabolic signals to a more than average extent. Examples of genes in those expression hotspots include opioid receptors (*Oprm/k)*, estrogen receptors (*Esr1/2*) and thyroid hormone receptors for the amygdala (*Thra/b*, **Figure 3B**). For quality control, after the selection of all metabolic receptors, the expression of all receptors within these expression hotspots was visually inspected in the original ISH data from the ABA. Due to experimental artifacts or annotation errors, we adjusted the expression location for 3 out of 46 gene-brain region combinations and excluded 8 out of 46 gene-brain region combinations (**Figure 2**). This resulted in a final list of 21 metabolic receptors for 16 factors that were expressed in the central fear circuitry to a more than average extent (**Supplementary Tables S1**, **S2**).

### Co-expression of Metabolic Receptors With *Crh* Reveals Potential Involvement With Fear Circuitries

**FIGURE 4:**
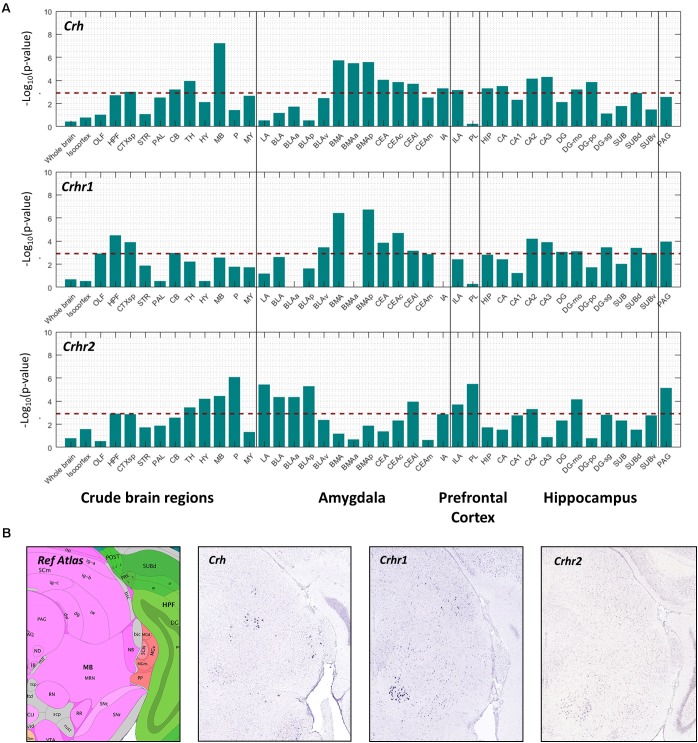
Co-expression of metabolic receptor genes with *Crh, Crhr1*, and *Crhr2*. **(A)** The strength of co-expression between the set of metabolic receptors and each of the seed genes *Crh*, *Crhr1*, and *Crhr2*. Each bar corresponds to –log_10_(*p*-value) (one-sided Wilcoxon rank-sum test) in 12 crude brain regions, amygdala, medial prefrontal cortex and hippocampus. The *horizontal dashed line* indicates the significance level at *p* = 0.05, Bonferroni-corrected for the number of structures. **(B)** Example sagittal sections from the Allen Brain Atlas (Image credit: Allen Institute), showing the reference atlas, the *in situ* hybridization of *Crh* (experiment number 292), *Crhr1* (experiment number 297), and *Crhr2* (experiment number 79907934) in the midbrain.

We further hypothesized that if a metabolic receptor is involved in the fear response, it may be coexpressed with other known fear modulators in the context of stress, i.e., corticotropin releasing hormone (*Crh*), corticotropin releasing hormone receptor 1 (*Crhr1*, predominant anxiogenesis) and corticotropin releasing hormone receptor 2 (*Crhr2*, anxiolysis) (Dedic et al., 2017). Pearson’s correlation coefficient (*r*) was used to correlate the spatial expression of metabolic receptor genes (**Table 1**) with seed genes *Crh, Crhr1, Crhr2*. A high positive correlation value implies that the expression profiles of the seed gene and the metabolic receptor gene are highly comparable, indicating co-expression. We assessed the spatial co-expression of metabolic receptor genes (**Table 1**) with seed genes *Crh, Crhr1, Crhr2* within each brain region separately. Within our predefined areas, the metabolic receptor gene set was significantly positively correlated with all seed genes in the hippocampal formation and in the cortical subplate (which comprises LA, BLA, BMA a.o, **Figure 4**). Of note, the highest correlation of metabolic receptors with *Crh* was found in the midbrain, where *Crh* is expressed in the superior colliculus and the nucleus ruber, areas not often associated with anxiety (**Figure 4B**). For the amygdaloid nuclei, the receptor gene set was mainly correlated with *Crh* and *Chr1* in the BMA and CEA, whereas in LA and BLA, the receptor gene set mainly correlated with *Crhr2* (**Figure 4A**). In the ILA, the metabolic receptor gene set was correlated with all seed genes; however, in PL the metabolic receptor gene set only significantly correlated with *Crhr2.* Throughout most hippocampal areas, the metabolic receptors were significantly correlated with the seed genes. In the PAG, the metabolic receptor set was significantly correlated with *Crhr1* and *Crhr2*, but not with *Crh.*

**FIGURE 5:**
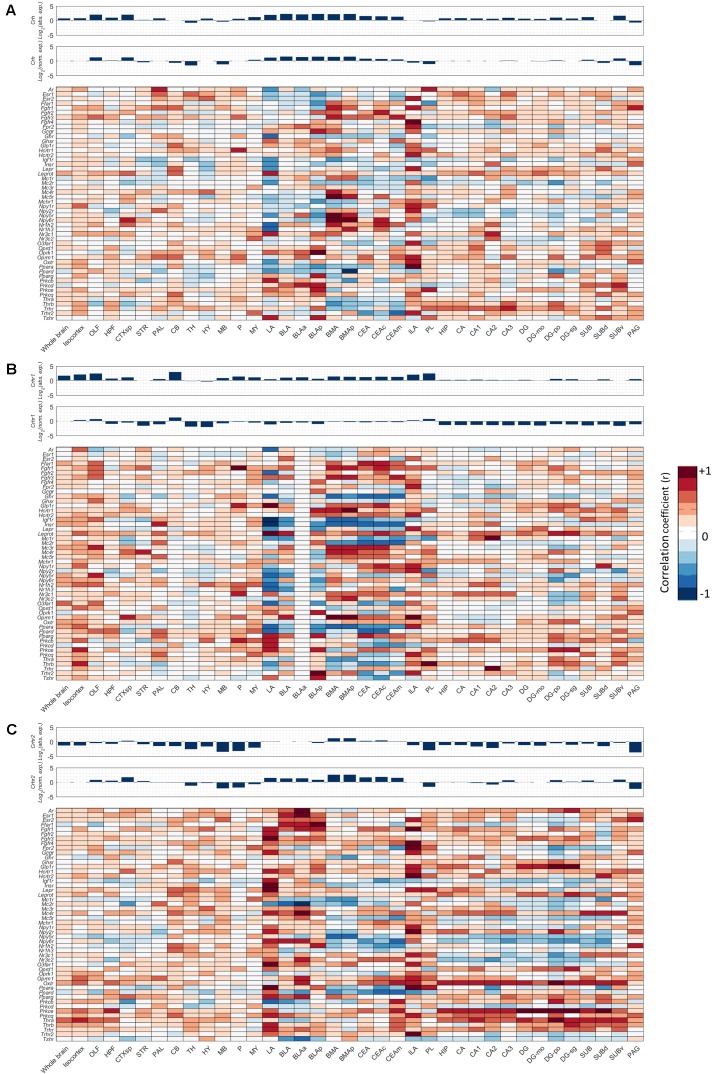
Co-expression of metabolic receptors with stress-associated genes *Crh, Crhr1*, and *Crhr2.* Pearson’s correlation coefficient was used to estimate the similarity between the 3D spatial expression profile of each metabolic receptor and the seed genes **(A)** *Crh*, **(B)** *Crhr1*, and **(C)** *Crhr2.* The expression profiles of the seed genes across the regions of interest are shown as bar plots above the co-expression heat maps, both absolute (Abs. exp.) (*top*) and normalized (Norm. exp.) to the average expression across the whole brain (*bottom*).

Next, we investigated for each individual metabolic receptor gene how they were correlated with the *Crh(R)* seed genes in 12 crude brain regions and the fear circuitry. HPT-axis receptor genes (*Thra*, *Thrb*), known as potent regulators of the fear response, were strongly positively correlated (*r* > 0.6) with *Crh*, *Crhr1*, and *Crhr2* in the ILA and *Thrb* also with *Crhr1* in the PL *r* = 0.84, **Figures 5A–C**. Furthermore, *Thra* was strongly positively correlated (*r* > 0.6) with *Crhr2* in the whole hippocampus, while *Thrb* positively correlated with *Crhr2* (*r* > 0.6) in the LA (**Figure 5C**). Altogether, these data point toward interactions between thyroid hormone and CRH signaling, both via interactions via the afferent hippocampus, PL and ILA, and via direct interactions with the amygdala.

### Putative Metabolic Interactors With the Fear Circuitry

**FIGURE 6:**
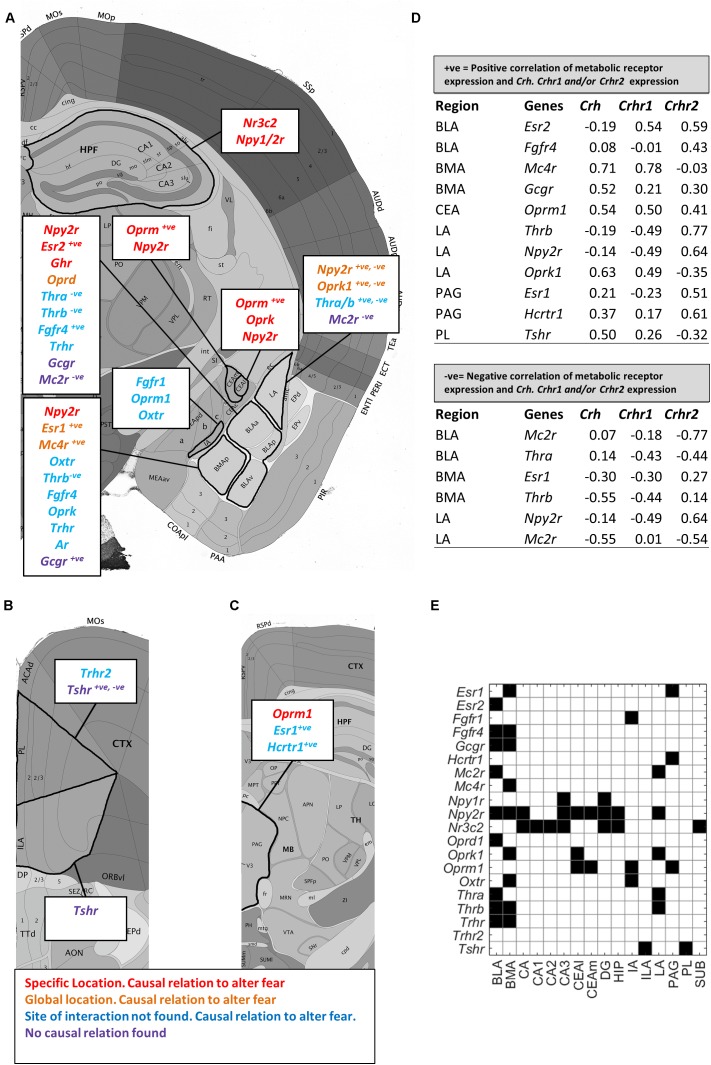
Enriched metabolic receptors in the central fear circuitry. Depicted metabolic receptor genes were selected for their high normalized expression [log_2_(normalized expression) > 2 for amygdala, hippocampus and medial prefrontal cortex; >1 for periaqueductal gray, **Figure 3**] in **(A)** hippocampus (HPF) and amygdala (CEA, LA, BLA, BMA, and IA), **(B)** medial prefrontal cortex (ILA, PL), and **(C)** periaqueductal gray (PAG). Color code indicates the findings of our literature review. *Red*: the metabolic factor of the receptor was found to causally alter fear at the location as indicated in the figure. *Orange*: the metabolic factor of the receptor was found to causally alter fear in the amygdala, but the responsible subnucleus is unknown. *Blue*: the metabolic factor of the receptor was found to causally alter fear, but the site of action is unknown. *Purple*: the metabolic factor of the receptor was not found to causally alter fear. +*ve* and –*ve*: the metabolic factor was highly positively or negatively correlated with *Crh, Crhr1*, or *Crhr2*. **(D)** Exact correlation values are indicated in the table. **(E)** Summary of all enriched metabolic receptors in the central fear circuitry (*black* = *enriched*).

In a next step, we reviewed literature on whether metabolic receptors within expression hotspots of the fear circuitry (**Supplementary Tables S1**, **S2**) directly regulated fear and whether the site of interaction within the fear circuitry had previously been described (**Figures 6A–C** and **Supplementary Table S5**). For the majority of metabolic factors, evidence was found they modulated the fear response, but where interactions took place was less often known. While thyroid hormones are known fear controllers, only little is known about the emotional effects of the upstream hormones of the HPT-axis. Based on high normalized receptor expression levels of thyroid stimulating hormone receptor (*Tshr*) and thyroid stimulating hormone receptor (*Trh*), thyroid stimulating hormone and thyroid releasing hormone are suggested to influence the emotional brain via projections from the PL and ILA or directly via the amygdala. Another remarkable finding is the particularly high normalized expression of the glucagon receptor (*Gcgr*) in the amygdala, while glucagon has never been described to influence fear previously.

We next deduced from **Figure 5** which of the overrepresented metabolic receptors were positively correlated with *Crh, Crhr1*, or *Crhr2* in the fear circuitry (**Figures 5**, **6A–D**, *marked as ‘*+*ve’*). As expected, some brain nuclei that are targeted by metabolic factors that are known to affect fear (like estrogens and opioids) also express *Crh, Crhr1*, or *Chrh2*. Interestingly, several receptors of metabolic factors that were *not* identified as fear modulators [e.g., glucagon receptor (*Gcgr*) in the BMA; thyroid stimulating hormone receptor (*Tshr)* in the PL] also showed an expression pattern similar to *Crh, Crhr1*, or *Crhr2.*

We additionally investigated which metabolic receptors were negatively correlated with the seed genes (**Figures 5**, **6A–D**, *marked as ‘-ve’*). While a high *positive* correlation indicates co-expression, a high *negative* correlation value suggests that the seed gene and the receptor gene are expressed in other cell populations. Some receptors were found to be coexpressed with one seed gene while being negatively correlated with the other (e.g., *Thrb* with *Crh* and *Crhr1*, respectively, in the BMA). Of note, receptors with (i.e., *Oprm1*) and without (i.e, *Mc2r*) a proven causal relation to fear were negatively correlated with seed genes. A graphic summary of all overrepresented metabolic receptors in the fear circuitry can be found in **Figure 6E**.

### Co-expression Analysis Reveals That Metabolic Factors Interact With Serotonergic and Noradrenergic Neurons

**FIGURE 7:**
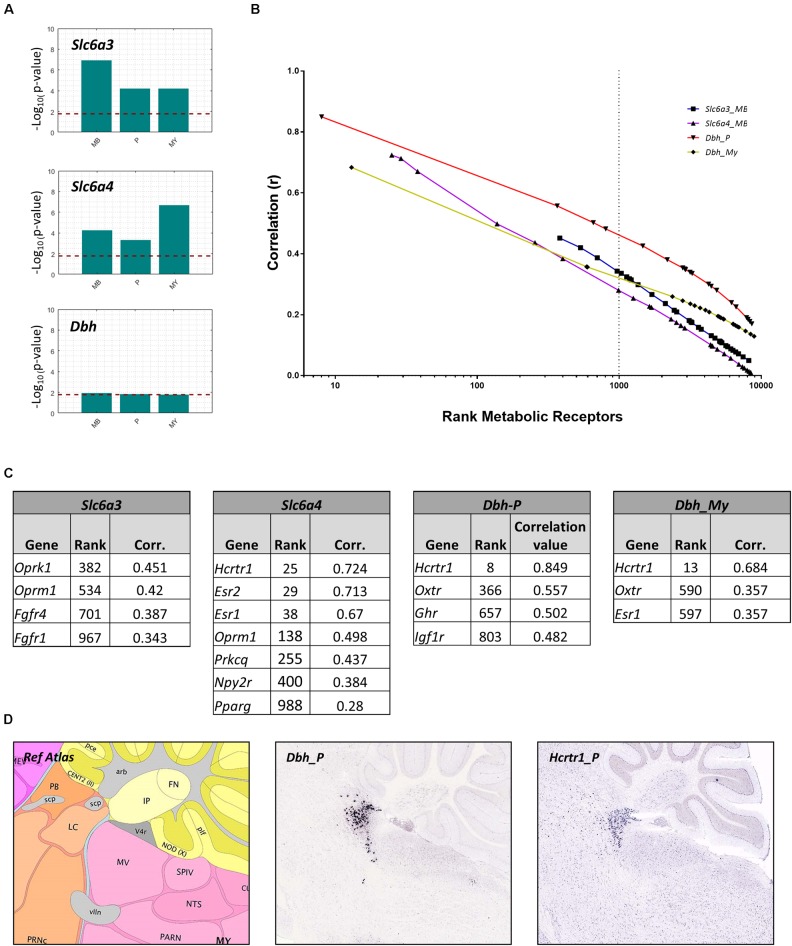
Co-expression of metabolic receptor genes with markers for neurons producing dopamine (*Slc6a3*), serotonin (*Slc6a4*), and noradrenalin (*Dbh*). **(A)** All bars indicate the –log_10_(*p*-value) of the one-sided Wilcoxon rank sum test used to assess the significance of the co-expression between the metabolic receptor gene set and each of the seed genes *Slc6a3*, *Sl6a4, Dbh* in midbrain (MB), pons (P), and medulla (MY). The horizontal dashed line indicates the significance level at *p* = 0.05, Bonferroni-corrected for the number of structures. **(B)** Correlation values of all genes included in ABA (including metabolic receptor genes) with seed genes (*Slc6a3, Sl6a4*, and *Dbh*) in relevant regions were ranked. Depicted are the ranks of the metabolic receptor genes, set out against respective correlation values. The genes left of the dashed line indicate the top 1,000 ranked metabolic receptor genes. **(C)** Correlation values of metabolic receptors among the top 1,000 ranked metabolic receptor genes. **(D)** Example sagittal sections from the Allen Brain Atlas, showing the reference atlas (*top*), the *in situ* hybridization of *Dbh* in Pons/Locus Coeruleus/Nucleus Tractus Solitarius (P/LC/NTS, respectively) (experiment 79913340) (*middle*), and Hcrtr1 in P/LC/NTS (experiment 1216) (*bottom*) (Image credit: Allen Institute).

In addition to directly targeting the fear circuitry, we hypothesized that metabolic factors may target monoaminergic projection areas to influence fear. Many monoaminergic brain nuclei were too small (i.e., smaller than 25 voxels in the ABA, **Table 2**) to evaluate receptor expression using a similar method as described above. Instead, we investigated whether metabolic receptors were coexpressed with markers for monoamine producing neurons. The receptor gene set was correlated with markers specific for DA producing cells (*Slc6a3)*, 5-HT producing cells (*Slc6a4)* and NE producing cells (*Dbh)*. To overcome the small size of the brain regions, correlations were calculated within parent brain regions in which monoaminergic nuclei were situated: the midbrain (MB) for ascending 5-HT/DA neurons (raphe nuclei and substantia nigra/ventral tegmental area, respectively), and for noradrenergic projections in the pons (locus coereleus, nucleus parabrachialis) and medulla (nucleus tractus solitarius). The metabolic receptor gene set was significantly correlated with *Slc6a3* and *Slc6a4*, but not with *Dbh* (**Figure 7A**).

**Table 2:** Brain regions included for *de novo* analysis.

Region acronym	Region name	Size (voxels)
CB	Cerebellum	3406
CTXsp	Cortical subplate	406
HPF	Hippocampal formation	1985
HY	Hypothalamus	1038
Isocortex	Isocortex	5759
MB	Midbrain	2626
MY	Medulla	1930
OLF	Olfactory areas	2376
P	Pons	1351
PAL	Pallidum	604
STR	Striatum	2690
TH	Thalamus	1393
BLA	Basolateral amygdalar nucleus, total	103
BLAa	Basolateral amygdalar nucleus, anterior part	57
BLAp	Basolateral amygdalar nucleus, posterior part	34
BLAv	Basolateral amygdalar nucleus, ventral part^∗^	12
BMA	Basomedial nucleus, total	78
BMAa	Basomedial nucleus, anterior part^∗^	18
BMAp	Basomedial nucleus, posterior part	60
CEA	Central amygdalar nucleus, total	93
CEAc	Central amygdalar nucleus, capsular part	38
CEAl	Central amygdalar nucleus, lateral part^∗^	16
CEAm	Central amygdalar nucleus, medial part	39
IA	Intercalated amygdalar nucleus^∗^	13
LA	Lateral amygdalar nucleus	36
ILA	Infralimbic cortex	131
PL	Prelimbic cortex	153
HIP	Hippocampal region	1234
CA	CA region total	817
CA1	CA1 region	443
CA2	CA2-region	43
CA3	CA3-region	331
DG	Dentate gyrus	405
DG-mo	Dentate gyrus, molecular layer	257
DG-po	Dentate gyrus, polymorph layer	47
DG-sg	Dentate gyrus, granule cell layer	101
SUB	Subiculum, total	293
SUBd	Subiculum, dorsal part	96
SUBv	Subiculum, ventral part	197
PAG	Periaqueductal Gray	432
LC	Locus Coereleus^∗^	3
NTS	Nucleus of the Solitary Tract	40
PB	Parabrachial Nucleus	68
Ramb	Midbrain raphe nuclei	66
IF	Interfascicular raphe nucleus^∗^	3
IPN	Interpeduncular nucleus^∗^	22
RL	Rostral linear raphe nucleus^∗^	8
CLI	Central linear raphe nucleus^∗^	12
DR	Dorsal raphe nucleus^∗^	21
SNc	Substantia nigra, compact part	30
SNl	Substantia nigra, lateral part^∗^	0
SNr	Substantia nigra, reticular part	94
VTA	Ventral tegmental area	52


*^∗^Brain region too small for coexpression analysis (<25 voxels).*

Next, we assessed whether the significant correlations between the metabolic receptor gene set and the seed genes were driven by few strongly correlated genes, or rather driven by many moderately correlated genes. We plotted the ranks of the metabolic receptor genes against their correlation coefficients and determined the top 1,000- highest ranked (**Figures 7B,C** and **Supplementary Table S3**). *Slca4* was correlated with the largest number of receptor genes (*n* = 7). This suggests that 5-HT is broadly sensitive to metabolic alterations, in accordance with literature. In contrast, *Dbh* correlated with only few receptor genes, but very strongly with the hypocretin receptor (*Hcrtr1*, **Figure 7C**). Indeed, *Hcrtr1* is highly colocalized with *Dbh* in the locus coeruleus, and to a lesser extent in the PB (**Figure 7D**) and orexin/hypocretin is known to be involved in anxiety states and arousal (Sears et al., 2013).

### Putative Metabolic Interactors With Monoamine Projection Regions

**FIGURE 8:**
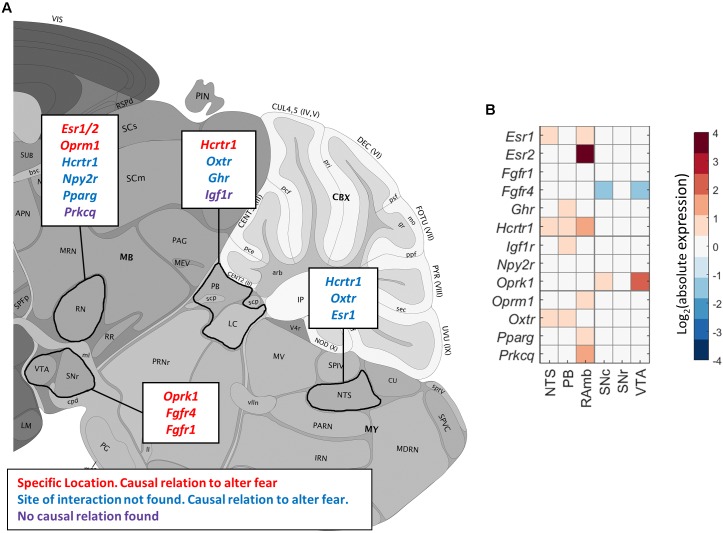
Enriched metabolic receptors in monoaminergic brain regions. **(A)** Depicted metabolic receptor genes were among the top 1,000 genes (**Figure 7C**) that are highly coexpressed with monoamine markers *Slc6a3* (in midbrain), *Slc6a4* (in midbrain), and *Dbh* (in pons and medulla). These monoamine markers are expressed in monoamine producing nuclei, i.e., *Slc6a3* in the ventral tegmental area (VTA) and the substantia nigra (SN), *Slc6a4* in the raphe nuclei of the midbrain (RN) and *Dbh* in the nucleus parabrachialis (PB), the locus coeruleus (LC), and the nucleus tractus solitarii (NTS). Color code indicates the findings of our literature review. *Red*: the metabolic factor of the receptor was found to causally alter fear at the location as indicated in the figure. *Blue*: the metabolic factor of the receptor was found to causally alter fear, but the site of action was unknown. *Green*: the metabolic factor of the receptor was not found to causally alter fear. **(B)** Absolute expression summary of all enriched metabolic receptors in monoaminergic brain regions.

We next verified the ABA ISH data for the identified candidate metabolic receptors in monoaminergic nuclei. This led to the exclusion of *Oprm1* expression in the SN and/or VTA (**Figure 2** and **Supplementary Tables S3**, **S4**). We found that the majority of the identified metabolic receptors in monoaminergic brain regions appeared to be known controllers of the fear response (**Figure 8** and **Supplementary Table S5**). Some metabolic factors, such as fatty acids and oxytocin, are known for their involvement in fear, but their sites of action are unidentified. Here we show that fatty acids might interact with the 5-HT system via receptor peroxisome proliferator-activated receptor gamma (*Ppar*γ) and that oxytocin might affect NE producing neurons as its receptor (*Oxtr*) is enriched in these regions. Of note, no direct relations to fear have been demonstrated yet for insulin-like growth hormone receptor (*Igf1r*) and the potential fatty acid binder Protein Kinase ζ (*Prkcq*) that are overrepresented in PB/LC and RN, respectively.

## Discussion

This study aimed to unveil metabolic signals that influence fear behavior and their cerebral targets. Expression data from the ABA showed that receptors of both known fear-associated metabolic factors, (e.g., NPY, estrogen and thyroid hormones) and potential new fear-related factors (e.g., glucagon) were overrepresented in the central fear circuitry. We observed no systematic enrichment for particular classes of metabolic signaling factors, such as hypothalamic peptides or adipokines. We additionally show that monoaminergic brain regions may be differentially responsive to metabolic changes. Serotonergic neurons seem broadly sensitive to metabolic signals, given the high number of colocalized metabolic receptor genes. Although dopamine is clearly linked to anticipation of, for example, food rewards, dopaminergic midbrain areas had a relatively low expression of metabolic receptors. The noradrenergic system appears to be specifically sensitive to modulation by hypocretin/orexin.

### Findings in Monoaminergic Brain Regions

The high hypocretin/orexin receptor 1 (*Hcrtr1)* expression in the LC is interesting in the light of regulation of fear and fear memories, especially because NA receptor stimulation in the amygdala, for example, is crucial for the formation of fear memories (Roozendaal et al., 2006). Hypothalamic orexin neurons send dense projections to the locus coeruleus (Sears et al., 2013). Via optogenetic stimulation, pharmacologic inhibition and disruption of these fibers, orexin was shown to promote fear memory consolidation via the LC. Transgenic mouse models additionally showed that *Hcrtr1*, and not *Hcrtr2* is important for the formation of threat memories (Soya et al., 2013) -, which is in line with our findings. The neurons in the LC that receive input from hypothalamic orexin fibers project to the LA to regulate fear (Soya et al., 2017). In the altered metabolic state of fasting, mice showed increased fear behavior as well as LA activity, which could be prevented by pharmacological inhibition of *Hcrtr1* in the LC.

Serotonergic neurons showed the broadest expression of metabolic receptors. This is in line with the current findings from literature, that report most frequently interactions between metabolic state and serotonergic transmission. This includes data from human studies (Muldoon et al., 2006) and findings after inducing obesity in rodents (Krishna et al., 2016). Serotonin can affect fear circuits either directly in the amygdala (Bocchio et al., 2016), or via other components of the fear circuitry (Burghardt and Bauer, 2013). The serotonin system is an example of a system with a two-way interaction, as it is in turn able to regulate feeding behavior and metabolic processes. Therefore the metabolic sensing by the serotonin system may also be part of metabolic feedback- or feedforward control loops (Xu et al., 2010; Jean et al., 2017).

### Findings in the Amygdala

In the LA and BLA, we found the metabolic receptor set to be significantly coexpressed with *Crhr2.* We and others found that *Crhr2* is modestly expressed in the BLA (Chalmers et al., 1995; Van Pett et al., 2000). Here we identified the estrogen receptor beta (*Esr2*) as one of the metabolic receptors that was highly coexpressed with *Crhr2* in the BLA. Estrogens are well known to alter fear behavior, and receptor type largely determines whether the effect is anxiogenic (*Esr1*) or anxiolytic (*Esr2*) (Borrow and Handa, 2017). Our data suggest that in particular contexts ER beta (*Esr2*) may also alter fear behavior by impacting on CRHR2 positive neurons, which are mainly involved in anxiolysis. Estrogens were also shown to be permissive for CRHR2 receptor expression in non-neuronal cells (Wang et al., 2012). Although estrogen levels rise during obesity (Brown, 2014), it is, to our knowledge, not known how estrogen receptor expression levels are affected in altered metabolic state. Other receptors with a high relative expression in the amygdala include the μ opioid receptor and the MC2 receptor, which we discuss below.

### Findings in Medial Prefrontal Cortex and Hippocampus

The PFC seemed relatively sensitive to thyroid hormone related receptors. The high correlation of metabolic receptors with *Crh(R)* in the ILA may be interesting in the pathogenesis of anxiety disorders. The ILA is thought to be involved in the conversion of a Pavlovian fear response toward a pathological anxiety disorder (Morgan et al., 1993). In a healthy fear response fear is easily extinguishable, while in anxiety disorders extinction is difficult to accomplish (LeDoux, 2000). Supporting the involvement of the ILA in this pathogenic conversion, damage to the medial prefrontal cortex in rats could convert easy-extinguishable fear to difficult-to-extinguish fear (Morgan et al., 1993). In the hippocampus, NPY receptors (Npyr1/2) stood out prominently. The Y1 receptors (Npyr1) and Y2 receptors (Npyr2) are the predominant NPY receptors in the central nervous system (Tasan et al., 2016). While Y1 receptors are post-synaptic and anxiolytic, Y2 receptors are pre-synaptic and anxiogenic, most likely because Y2 receptors inhibit the release of anxiolytic NPY (Wu et al., 2011). In acute situations, the orexigenic and anxiolytic effects of NPY support the relation between hunger and a reduction of fear. However, increased NPY-signaling also drives the hyperphagia during obesity, which would contradict the association between obesity and anxiogenesis.

### Findings in the Periaqueductal Gray

In the PAG, the opioid receptor mu (*Oprm1*) was found to be highly expressed, which most likely is involved in fear and analgesia. The systems for nociception and fear are closely and bidirectionally connected. If a neutral stimulus predicts a nociceptive stimulus, the neutral stimulus will acquire the ability to activate the defensive system, and becomes a conditioned stimulus (Fanselow, 1986). In reverse, the defensive system activates analgesic processes which supports defensive behavior. The periaqueductal gray is a major integration site of nociceptive and fear stimuli and hence is an important driver of fear-induced analgesia (Behbehani, 1995). Indeed, *Oprm1* agonist morphine injected into the PAG decrease fear and promotes analgesia, while morphine withdrawal is associated with anxiogenesis (De Ross et al., 2009; Halladay and Blair, 2012; Twardowschy and Coimbra, 2015). In humans, obesity is associated with decreased *Oprm1* availability in the brain, which would support the notion that obesity raises fear levels (Karlsson et al., 2015; Pitman and Borgland, 2015).

### Glucagon Is a Novel Metabolic Factor That May Influence Fear

Unexpectedly, the glucagon receptor (*Gcgr*) was overrepresented in the BLA and BMA, while it is unknown to have any effects on emotion regulation. Radioactive-labeling studies have shown that glucagon indeed binds to glucagon receptors in the amygdala (Hoosein and Gurd, 1984). We hypothesize that the glucagon receptors in the BLA and BMA may have a role in fear, or in food intake, or both. Previous work shows that central glucagon acts as anorectic hormone and regulates peripheral glucose levels, although this is mostly driven by non-limbic systems (Abraham and Lam, 2016). Given that the amygdala also regulates food intake, as for example evident in appetitive fear conditioning experiments (Petrovich, 2013), we hypothesize that the anorectic effects of glucagon may also be mediated via the amygdala. Indeed, subcutaneously injected glucagon acted in concert with GLP-1 to reduce food intake, and induced a similar pattern of c-fos expression in the central nucleus of the amygdala (Parker et al., 2013). Supportive of a potential fear modulating role for glucagon, type II diabetes patients, which have both elevated insulin and glucagon levels (Cryer, 2012), have an increased risk to develop anxiety disorders (OR = 1.25 (CI 1.10–1.39) [18]). The high co-expression with *Crhr1* and stimulatory nature of the glucagon receptor suggest that like NPY, glucagon is a signal for a negative energy balance that affects in parallel food intake and anxiety.

### ACTH Is a Novel Metabolic Factor That May Influence Fear

We additionally found that in the BLA and LA, the cognate ACTH receptor (Mc2r) was relatively highly expressed. From studies on ACTH fragments it is known that the N-terminal part of ACTH influences learning, motivation and social behavior (de Wied, 1990) – yet, to our knowledge very few studies on the neuroactive effects of *full* ACTH protein have ever been performed, once the activity of ACTH fragments was discovered (De Wied, 1965; Greven and de Wied, 1967). Assuming that ACTH is the main ligand for MC2 receptors within the brain, it is likely that it promotes stress responses and may – like CRF – be stimulatory for anxiety and fear.

### Novel Cerebral Targets for the HPT-axis That May Control Fear Behavior

Besides the identification of metabolic factors that may regulate fear, the data set also allows for identification of novel sites of action for known fear-regulating metabolic factors. The HPT-axis is for example known to affect fear behavior, but it is relatively unknown where those interactions take place. Here we show that the amygdala may be a major site of action, as in the BLA, BMA, and LA expresses the thyroid hormone receptors (*Thra/b)* to a relatively high level. In addition, the medial prefrontal cortex may be a major interaction site, as *Tshr* and *Trhr* are overrepresented in these brain regions. We additionally hypothesize that *Thra/b* and *Tshr* may interact with the CRH/urocortin system, as they have a similar expression profile as *Crh* and its receptors. As for ACTH, the effects of TSH within the brain are poorly characterized, but – in line with our selection criteria – there is functional evidence for TSH effects on the rodent brain in relation to food intake (Burgos et al., 2016). The co-expression data suggest that these effects may be extend to include fear regulation.

### Novel Cerebral Targets for Integration of Metabolic Signaling and Fear Behavior

We additionally found a novel neuroanatomical site that could be important in the integration of metabolic signals and fear. Co-expression with fear-associated genes *Crh, Crhr1*, and *Crhr2* across crude brain regions revealed that the metabolic receptor set correlated mostly in areas that are not often associated with anxiety, i.e., midbrain CRH expressing neurons, as present in the superior colliculus and nucleus ruber. Studies in amphibians suggest that CRH-neurons in the superior colliculus regulate visually guided feeding (Prater et al., 2017). As the superior colliculus is part of the fear-related subcortical visual pathway, our findings may point to the visual processing of threats and fear.(Carr et al., 2013).

### Technical Limitations

There are some technical limitations to our approach. First, the high throughput ISH data of the ABA are not always of high quality, which can lead to false negative (in case of failed ISH) and false positive (in case of mislabelling of a brain area) results. To overcome the latter, all our findings were verified by visual inspection of the source ISH data. Second, the importance of *high* values of normalized expression values may be overestimated if the absolute expression is low (e.g., *Trhr2*). Conversely, the importance of *low* values of normalized expression values may be underestimated (e.g., *Nr3c1, Igf1r*), especially if a gene is generally highly expressed throughout the brain. Lastly, correlation values tend to be higher in smaller regions, such as for the LA region. Nevertheless, the comparison of correlation values in the LA (36 voxels) with other brain regions of similar size, i.e., BLAp (34 voxels) and CEAc (38 voxels) suggests that increased correlation values in the LA most likely reflect a biological phenomenon.

### Biological Limitations

The *post hoc* observation of a large number of known fear-associated factors in our set of selected metabolic receptors supports its validity. However, we may have included non-specific metabolic receptors, such as fatty-acid sensor protein kinase C (*Prkcb/d/e/q*) that also exerts a wide variety of other, more general cellular actions. In addition, it is not always conclusive whether a given factor can cross the blood–brain-barrier (BBB). This was especially the case for ACTH, as barrier penetrance was primarily investigated with ACTH analogs and fragments (Reul et al., 1988; de Wied, 1990; Burchuladze et al., 1994; Vasadze et al., 2007). However, the overrepresented MC2 receptors in amygdala and medial PFC suggest that *Mc2r* at least exerts some central functions. Furthermore, we considered potential direct at-site interactions, hereby potentially overlooking factors that influence the central nervous system via other ways (e.g., gut-brain axis). As we looked at responsiveness to metabolic factors, we exclusively focussed on receptor expression, although we are aware that genes encoding for metabolic factors or factor-producing enzymes can be of biological significance. For the literature review, we had stringent inclusion criteria to exclusively select causal studies, which might have led to ignoring associations without a causal relation (e.g., *Igf1r*; Baldini et al., 2013; Brambilla et al., 2018). We have not validated co-expression data with immunofluorescence given the vast number of options, but do suggest this as a first step for follow up experiments. As a final remark, interpretations about novel fear-modulating factors should be carefully made as included brain regions also exert other brain functions that are unrelated to fear.

### Future Research

The resulting data present several hypotheses for future fear research. The suggested role in fear regulation for some metabolic factors should be validated *in vivo*, preferably with microinjection studies using (ant)agonists, or other targeted approaches. For each metabolic factor the directionality of effects on fear behavior could be predicted (anxiolysis/genesis), potentially in the context of a positive or negative energy balance. Yet, this approach still needs to overcome a number of obstacles. For instance, metabolic state –especially obesity- can alter the permeability of the blood–brain-barrier (Banks, 2008; Urayama and Banks, 2008). In addition, some peripheral factors are also centrally produced (e.g., GLP-1), which makes the ‘metabolic load’ that is seen by a brain nucleus difficult to predict (Cabou and Burcelin, 2011). Further predictions would be based on whether cognate receptors are stimulatory or inhibitory, which is complex for nuclear receptors as they can both activate and repress gene transcription (e.g., *Thra/b*, *Nr3c1*, *Er1/2*). Finally, receptors are expressed at inhibitory or excitatory neurons in a stimulatory or inhibitory (sub)nucleus. To gain more insight which neuronal cell populations express metabolic receptors, the metabolic receptor gene set might be correlated with *Slc6a1/Gat1* (GABAergic neuron marker) and *Slc17a1/Vglut1* (Glutamatergic neuron marker).

## Conclusion

All components of the brain fear network seem responsive to metabolic factors, but with a degree of specificity. Similar approaches on available human brain transcriptomes [e.g., from the Allen Human Brain Atlas (Hawrylycz et al., 2012)] may help to reveal evolutionary conservation of the described patterns of expression. It would additionally be important to validate gene interactions at the protein level (Sharma et al., 2015). The current study emphasizes the role of different metabolic factors in shaping human brain responsiveness and may be used as a rich dataset to guide new approaches to study the interactions between metabolic state and fear.

## Author Contributions

LK and OM conceived the setup and wrote the paper. AM and MR conceived the expression analysis. AM performed the bioinformatics analysis. MB performed the *post hoc* validation of results and the literature review. All authors discussed the analyses and manuscript.

## Conflict of Interest Statement

The authors declare that the research was conducted in the absence of any commercial or financial relationships that could be construed as a potential conflict of interest.
