# Supplementary material for: How Metabolic State May Regulate Fear: Presence of Metabolic Receptors in the Fear Circuitry
Source: Front Neurosci. 2018 Aug 27;12:594. doi: 10.3389/fnins.2018.00594 (PMC6119828; doi:10.3389/fnins.2018.00594)
Supplement: Supplementary file 1 [file Data_Sheet_1.docx]

How metabolic state may regulate fear:
Running title: Metabolic receptors in the fear circuitry

Running title: Metabolic Receptors in the Fear Circuitry

Lisa L. Koorneef^1,2^, Marit Bogaards^1,2^, Marcel J.T. Reinders^3,4^, Boudewijn P.F. Lelieveldt^4,5^, Onno C. Meijer^1,2,6*^ Ahmed Mahfouz^3,4*^

# Correspondence: Lisa Koorneef – [L.L.Koorneef@lumc.nl](mailto:L.L.Koorneef@lumc.nl)

*Shared last authorship

**Contents:**

# Supplementary Data

# 1.1 Supplementary Figures

# 1.2 Supplementary Tables

**1.0 Supplementary Data**

**1.1 Supplementary Figures**

**
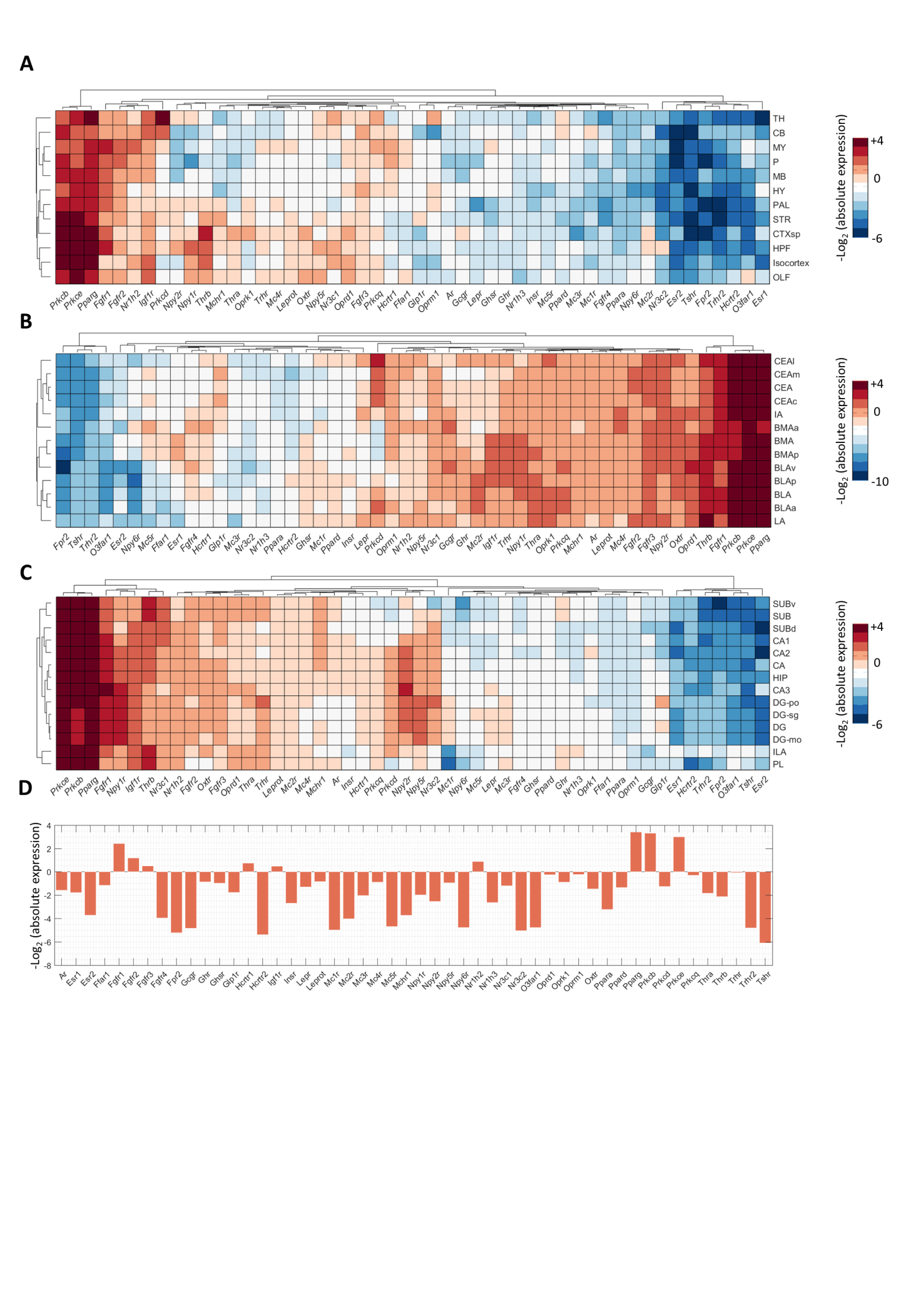
**

**Fig. S1- Absolute expression summary of metabolic receptor genes in fear-related brain regions.** Hierarchically clustered absolute expression energies across the whole brain in A) 12 crude brain regions B) amygdala C) hippocampus and medial prefrontal cortex and D) periaqueductal gray.

**1.2 Supplementary Tables**

**Supplementary Information**

**Table S1- Peripheral metabolic receptors that are overrepresented in the central fear circuitry.** Receptors were within expression hotspots in the fear circuitry, which are regions where receptor expression is particularly high (-log_2_(normalized expression) > 2 for amygdala, hippocampus and PFC; >1 for PAG, which is less neuron dense).

| **Peripheral metabolic factors** | | |
| --- | --- | --- |
| **Central fear circuitry** | | |
| **Factor** | **Receptor** | **Brain region** |
| ACTH | *Mc2r* | BLA |
| ACTH | *Mc2r* | LA |
| Estrogens | *Esr1* | BMA |
| Estrogens | *Esr1* | PAG |
| Estrogens | *Esr2* | BLA (was IA) |
| Fatty acids | *Fgfr1* | IA |
| FGF-21 | *Fgfr4* | BLA |
| FGF-21 | *Fgfr4* | BMA |
| GLP-1 | *Glp1r* | HIPPO |
| Glucagon | *Gcgr* | BLA |
| Glucagon | *Gcgr* | BMA |
| Glucocorticoids | *Nr3c2* | HIPPO |
| Growth Hormone | *Ghr* | BLA |
| Thyroid hormones | *Thra* | BLA |
| Thyroid hormones | *Thra* | LA |
| Thyroid hormones | *Thrb* | BLA |
| Thyroid hormones | *Thrb* | BMA |
| Thyroid hormones | *Thrb* | LA |
| TSH | *Tshr* | DmPFC |
| TSH | *Tshr* | VmPFC |
| Estrogens | *Esr1* | BLA |
| Estrogens | *Esr2* | BMA |
| FGF-21 | *Fgfr1* | BMA |
| Leptin | *Lepr* | LA |
| Androgens | *Ar* | IA |
| Fatty acids | *Fpr2* | HIPPO |

***Grey:*** Receptor expression in a brain region could not be confirmed with visual inspection of ISH data.

***Grey:*** Receptor expression in a brain region could not be confirmed with visual inspection of ISH data.

**Table S2- Central metabolic receptors that are overrepresented in the central fear circuitry.** Receptors were within expression hotspots in the fear circuitry, which are regions where receptor expression is particularly high (-log_2_(normalized expression) > 2 for amygdala, hippocampus and PFC; >1 for PAG, which is less neuron dense).

| **Central metabolic factors** | | |
| --- | --- | --- |
| **Central fear circuitry** | | |
| **Factor** | **Receptor** | **Brain region** |
| MSH | *Mc4r* | BMA (was IA) |
| Neuropeptide Y | *Npy1r* | HIPPO |
| Neuropeptide Y | *Npy2r* | BLA |
| Neuropeptide Y | *Npy2r* | BMA |
| Neuropeptide Y | *Npy2r* | CEA |
| Neuropeptide Y | *Npy2r* | HIPPO |
| Neuropeptide Y | *Npy2r* | LA |
| Opioids | *Oprd1* | BLA |
| Opioids | *Oprk1* | BMA (was BLA) |
| Opioids | *Oprk1* | CEA |
| Opioids | *Oprk1* | LA |
| Opioids | *Oprm1* | CEA |
| Opioids | *Oprm1* | IA |
| Opioids | *Oprm1* | PAG |
| Orexin /Hypocretin | *Hcrtr1* | PAG |
| Oxytocin | *Oxtr* | BMA |
| Oxytocin | *Oxtr* | IA |
| TRH | *Trhr2* | PL |
| Orexin /Hypocretin | *Hcrtr2* | BLA |
| MSH | *Mc4r* | BMA |
|  |  |  |

***Grey:*** Receptor expression in a brain region could not be confirmed with visual inspection of ISH data.

**Table S3- Peripheral metabolic receptors that are overrepresented in monoaminergic nuclei.** Metabolic receptor genes were among the top 1,000 genes that are highly coexpressed with monoamine marker *Slc6a3* (SN/VTA), *Sl6a4* (RN) and *Dbh* (LC/PB or NTS).

| **Peripheral metabolic factors** | | |
| --- | --- | --- |
| **Monoaminergic nuclei** | | |
| **Factor** | **Receptor** | **Brain region** |
| Estrogens | *Esr1* | NTS |
| Estrogens | *Esr1* | RN |
| Estrogens | *Esr2* | RN |
| Fatty acids | *Pparg* | RN |
| Fatty acids | *Prkcq* | RN |
| FGF-21 | *Fgfr1* | SN/VTA |
| FGF-21 | *Fgfr4* | SN/VTA |
| Growth Hormone | *Ghr* | LC/PB |
| IGF-1 | *Igf1r* | LC/PB |

**Table S4- Central metabolic receptors that are overrepresented in monoaminergic nuclei.** Metabolic receptor genes were among the top 1,000 genes that are highly coexpressed with monoamine marker *Slc6a3* (SN/VTA), *Sl6a4* (RN) and *Dbh* (LC/PB or NTS).

| **Central metabolic factors** | | |
| --- | --- | --- |
| **Monoaminergic nuclei** | | |
| **Factor** | **Receptor** | **Brain region** |
| Neuropeptide γ | *Npy2r* | RN |
| Orexin /Hypocretin | *Hcrtr1* | LC/PB |
| Orexin /Hypocretin | *Hcrtr1* | NTS |
| Orexin /Hypocretin | *Hcrtr1* | RN |
| Oxytocin | *Oxtr* | LC/PB |
| Oxytocin | *Oxtr* | NTS |
| Opioids | *Oprk1* | SN/VTA |
| Opioids | *Oprm1* | RN |
| Opioids | *Oprm1* | SN/VTA |

***Grey:*** Receptor expression in a brain region could not be confirmed with visual inspection of ISH data.

T**able S5-Causal relationships between ligands of metabolic receptors and fear.**

| Candidate metabolic factor | Site of interaction | Technique | Species | Directio-nality | References |
| --- | --- | --- | --- | --- | --- |
| Androgens | Unknown | Testicular feminization mutation of the AR | Rats | Anxiogenic | Zuloaga *et al.* 2011 |
| Estrogens | Unknown | Erbeta KO | Mice | Anxiolytic | Krezel *et al.* 2001, Imwalle *et al.* 2005, Oyola *et al.* 2012 |
|  | RN | Microinj. Estrodiol benzoate in medial RN | Rats | Anxiolytic | Andrade *et al.* 2005, Andrade *et al.* 2009 |
|  | BLA | Overexpression ERGR | Rats | Anxiolytic | Mitra *et al.* 2010 |
|  | Whole amygdala | Microinj. estrogen | Rats | Anxiolytic | Frye *et al.* 2004 |
|  | PAG | *Esr1* polymorphism | Humans | Anxiolytic/genic | Comings *et al.* 1999, Tiemeier *et al.* 2005, Ryan *et al.* 2011 |
| Fatty acids | Unknown | Pparg KO | Mice | Anxiolytic | Domi *et al.* 2016 |
| FGF21 | BLA | FGF2 microinj. | Rats | Anxiolytic | Graham *et al.* 2011 |
|  | SN/VTA | Fgfr1 KO | Mice | Anxiolytic | Brooks *et al.* 2014 |
|  | Unknown | Fgfr1 KO; FGF2KO | Mice | anxiolytic | Brooks *et al.* 2014, Salmaso *et al.* 2016s |
| Glucocorticoids | Hippocampus | Forebrain MR KO overexpression, microinfusion MR antagonists | Mice, rats | Anxiolytic/genic | Smythe *et al.* 1997, Rozeboom *et al.* 2007, McEown *et al.* 2011, Albrecht *et al.* 2013 |
| GLP-1 | Unknown | ICV GLP-1, GLP1 overexpression | Rats | Anxiolytic/genic | Moller *et al.* 2002, Gulec *et al.* 2010, Sharma *et al.* 2015, Anderberg *et al.* 2016 |
| Growth Hormone | BLA | Viral overexpression Ghr | Rats | Anxiogenic | Meyer *et al.* 2014, Gisabella *et al.* 2016 |
| Melanocortin system | Whole amygdala | Microinj into amygdala of Mc4R ant-agonist, | Rats | Anxiogenic | Kokare *et al.* 2005 |
|  | Unknown | ICV Mc4r antagonist, intranasal Mcr Antagonist | Rats | Anxiogenic | Shimazaki *et al.* 2005, Kokare *et al.* 2010, Serova *et al.* 2013 |
| Neuropeptide gamma | Hippocampus | miccroinj. NPY, Npy1r overexpression | Rats, mice | Anxiolytic | Smialowska *et al.* 2007, Olesen *et al.* 2012, Christiansen *et al.* 2014 |
|  | CEA | Npy2r specific KO/overexpression in CEA, KO | mice | Anxiolytic/genic | Lyons *et al.* 2010, Tasan *et al.* 2010, Verma *et al.* 2015 |
|  | Whole amygdala | microinj into amygdala, lentiviral NPY overexpression | Rats, mice | Anxiolytic | Kokare *et al.* 2005, Christiansen *et al.* 2014 |
|  | BLA | lentiviral microinj NPY, Npy2r KO; microinj Npyr1/2 agonist, | Rats | Anxiogenic | Sajdyk *et al.* 1999, Sajdyk *et al.* 2002, Tasan *et al.* 2010 |
|  | Unknown | ICV Npy, ICV Npy1r antagonist, Npy1r KO, ICV Npy2r agonist | Mice | Anxiolytic/genic | Karlsson *et al.* 2008, Morales-Medina *et al.* 2012, Desai *et al.* 2014, Lach *et al.* 2016 |
| Opioids | SN/VTA | Microinj. antagonist | Rats | Anxiogenic | Grahn *et al.* 1999 |
|  | BLA | Microinj KOR antagonist | Mice, Rats | Anxiogenic | Bruchas *et al.* 2009, Knoll *et al.* 2011 |
|  | CEA | Microinj. KOR antagonist | Rats | Anxiolytic/genic | File *et al.* 1979, Zarrindast *et al.* 2008, Randall-Thompson *et al.* 2010, Knoll *et al.* 2011, Poulin *et al.* 2013 |
|  | PAG | Microinj agonist/antagoist | Rats | Anxiolytic | Halladay *et al.* 2012, Silva *et al.* 2014 Brandao 1993, Motta *et al.* 1993, Anseloni *et al.* 1999, De Ross *et al.* 2009 |
|  | RN | Microinj DRN | Rats | Anxiogenic | Grahn *et al.* 1999 |
|  | SN/VTA | microninj mu/deltal/kappa opioid agonists or enkephalin metabolism inhibitor | Rats | Anxiogenic | Calenco-Choukroun *et al.* 1991 |
|  | Whole amygdala | Kappa-opioid rec ant-agonist microinj.; fMRI after naloxone | Mice, humans | Anxiolytic/genic | Good *et al.* 1995, Narita *et al.* 2006, Eippert *et al.* 2008, Smith *et al.* 2012 |
|  | Unknown | dynorphin KO, polymorphism dynorphin gene, Oprm KO, Oprd1 KO, | Rats, humans, mice | Anxiolytic/genic | Filliol *et al.* 2000, Sanders *et al.* 2005, Bilkei-Gorzo *et al.* 2012 |
| Orexin/hypocretin | LC/PB | Hcrtr1 KO, Hcrtr2 KO, microinj. Hcrtr1 (ant)agonist | Mice | Anxiolytic/genic | Sears *et al.* 2013, Soya *et al.* 2013 |
|  | Unknown | Orexin KO, Orexin ICV, Hcrtr1 KO | Mice, Rats | Anxiogenic | Suzuki *et al.* 2005, Abbas *et al.* 2015, Khalil *et al.* 2017 |
| Oxytocin | Unknown | ICV oxytocin, oxytocin KO | Mice | Anxiolytic/genic | Peters *et al.* 2014 |
| Thyroid Hormones | Unknown | Thra impairing mutation or KO, hypothroid models, Thra/b KO | Mice | Anxiolytic/genic | Guadano-Ferraz *et al.* 2003, Venero *et al.* 2005, Wilcoxon *et al.* 2007, Pilhatsch *et al.* 2010, Vasudevan *et al.* 2013, Buras *et al.* 2014, Yu *et al.* 2015, Richard *et al.* 2017 |
| TRH | Unknown | intranasal/ICV TRH,TRHR1/2 KO | Rats, mice | Anxiolytic/genic | Zeng *et al.* 2007, Gutierrez-Mariscal *et al.* 2008, Sun *et al.* 2009, Vinogradova *et al.* 2014, Vogel *et al.* 1980, Thompson *et al.* 2000 |
